# Assessing the intracellular primary metabolic profile of *Trichoderma reesei and Aspergillus niger* grown on different carbon sources

**DOI:** 10.3389/ffunb.2022.998361

**Published:** 2022-09-27

**Authors:** Gustavo Pagotto Borin, Juliana Velasco de Castro Oliveira

**Affiliations:** ^1^Brazilian Biorenewables National Laboratory (LNBR), Brazilian Center for Research in Energy and Materials (CNPEM), São Paulo, Brazil; ^2^Graduate Program in Genetics and Molecular Biology, Institute of Biology, University of Campinas (UNICAMP), São Paulo, Brazil

**Keywords:** *T. reesei*, *A. niger*, sugarcane bagasse, metabolism, metabolomics

## Abstract

*Trichoderma reesei* and *Aspergillus niger* are efficient biological platforms for the production of various industrial products, including cellulases and organic acids. Nevertheless, despite the extensive research on these fungi, integrated analyses of omics-driven approaches are still missing. In this study, the intracellular metabolic profile of *T. reesei* RUT-C30 and *A. niger* N402 strains grown on glucose, lactose, carboxymethylcellulose (CMC), and steam-exploded sugarcane bagasse (SEB) as carbon sources for 48 h was analysed by proton nuclear magnetic resonance. The aim was to verify the changes in the primary metabolism triggered by these substrates and use transcriptomics data from the literature to better understand the dynamics of the observed alterations. Glucose and CMC induced higher fungal growth whereas fungi grown on lactose showed the lowest dry weight. Metabolic profile analysis revealed that mannitol, trehalose, glutamate, glutamine, and alanine were the most abundant metabolites in both fungi regardless of the carbon source. These metabolites are of particular interest for the mobilization of carbon and nitrogen, and stress tolerance inside the cell. Their concomitant presence indicates conserved mechanisms adopted by both fungi to assimilate carbon sources of different levels of recalcitrance. Moreover, the higher levels of galactose intermediates in *T. reesei* suggest its better adaptation in lactose, whereas glycolate and malate in CMC might indicate activation of the glyoxylate shunt. Glycerol and 4-aminobutyrate accumulated in *A. niger* grown on CMC and lactose, suggesting their relevant role in these carbon sources. In SEB, a lower quantity and diversity of metabolites were identified compared to the other carbon sources, and the metabolic changes and higher xylanase and pNPGase activities indicated a better utilization of bagasse by *A. niger*. Transcriptomic analysis supported the observed metabolic changes and pathways identified in this work. Taken together, we have advanced the knowledge about how fungal primary metabolism is affected by different carbon sources, and have drawn attention to metabolites still unexplored. These findings might ultimately be considered for developing more robust and efficient microbial factories.

## Introduction

Filamentous fungi are biological systems used for industrial production of diverse products, including enzymes, organic acids, and secondary metabolites (Frisvad et al., 2018; Wösten, 2019). Among ascomycetes, *Trichoderma reesei* stands out as the most efficient producer of cellulases for the conversion of lignocellulosic biomass into bioethanol and chemicals (e.g., xylitol and carboxylic acids) (Druzhinina and Kubicek, 2017a; Champreda et al., 2019), while *Aspergillus niger* is widely used for the production of β-glucosidase (Lopes et al., 2018) and citric acid (Tong et al., 2019).

Both fungi have been subject to numerous studies involving genetic and metabolic engineering (Fonseca et al., 2020; Kun et al., 2020; Wang et al., 2018b; Xie et al., 2020; Xu et al., 2020), development of new gene expression platforms and artificial transcriptional regulators (Zhang et al., 2018; Mojzita et al., 2019; Meng et al., 2020; Zhang et al., 2020), and protein production (Dey et al., 2018; Díaz et al., 2020). Nevertheless, despite the great advances in the past decades, integrated analyses of omics-driven approaches correlated with the phenotype observed are scarce. In this sense, Rush et al. (2021) proposed a roadmap for exploring the potential of *Trichoderma* species at producing natural products, since genomic mining until the characterization of secondary metabolites by metabolomics and other approaches.

Unlike the large number of articles addressing the characterization of cellulases and other enzymes, production of secondary metabolites, signaling transduction pathways, and heterologous expression in *T. reesei*, few studies assessed its metabolism at a comprehensive metabolic level, barely using different carbon sources. For example, Jouhten et al. (2009) investigated the metabolic profile of *T. reesei* strains QM6a and the carbon catabolite de-repressed *Δcre1* grown on ^13^C-labelled glucose and sorbitol, being the last supplemented or not with the cellulase inducer sophorose. According to the nuclear magnetic resonance (NMR) analysis, supplementation of sophorose in ^13^C-labelled sorbitol did not induce significant changes in the flux ratios compared to the control, and *T. reesei* forwarded the carbon flux to the respiratory metabolism (i.e., the tricarboxylic acid (TCA) cycle) regardless of the strain or carbon source used. The metabolism of *T. reesei* was also evaluated *in silico* by constructing and simulating metabolic reactions under different conditions. Pitkänen et al. (2014) developed a computational method for constructing metabolic networks of 49 fungi, including *T. reesei* and *A. niger*, based on a genome-scale reconstruction. The same research group applied the metabolic model to *T. reesei* cultivated in a bioreactor using cellobiose as the carbon source (Pakula et al., 2016). The metabolic model was then refined with some improvements – such as the inclusion of a biomass equation based on experimental data and the use of reaction directions to fulfill gaps – and according to the authors, it is ready to be used for simulating protein production in *T. reesei* (Castillo et al., 2016).

Similar to *T. reesei*, studies investigating the metabolic profile of *A. niger* grown on different carbon sources are not numerous. Ruijter and Visser (1996) conducted a pioneering work to quantify the intermediate metabolites of the glycolytic pathway in *A. niger* grown on glucose. Next, Meijer et al. (2007) assessed the central metabolism of *A. niger* altered by different micro-aeration rates aiming at succinate production. Using xylose as the carbon source, they observed an increase in sugar alcohols and organic acids (succinate, fumarate, and malate) in the extracellular space (Meijer et al., 2007). In addition, this group examined the metabolic and transcriptomic changes induced by the overexpression of isocitrate lyase *icl* gene in *A. niger* (Meijer et al., 2009). They expected a decrease in the TCA cycle flux and higher succinate and malate production through the glyoxylate shunt pathway. Instead, *icl* overexpression increased the production of fumarate, but not of succinate. Addition of malonate, which is a succinate dehydrogenase inhibitor, was also evaluated aiming to increase succinate production. It enhanced citrate and oxalate production in the wild-type strain, while malate increase and no succinate were observed in the recombinant strain. The differences noted were attributed to the transport mechanisms of the organic acids, as well as different regulations of metabolic pathways and activity of their enzymes (Meijer et al., 2009).

Driouch et al. (2012) assessed the metabolic changes of a recombinant *A. niger* strain expressing a fructofuranosidase encoding gene grown on ^13^C-labelled glucose through *in vivo* metabolic flux analysis combined with gas chromatography coupled to mass spectrometry (GC-MS). They verified a change in the flux distribution towards the pentose phosphate pathway (PPP), activation of the mitochondrial malic enzyme, and a reduced flux through TCA in the recombinant strain. The high demand of NADPH for the fructofuranosidase biosynthesis might have contributed to the greater flux towards the PPP and malic enzyme activity in the recombinant strain, as both pathways generate the cofactor and could compensate for the reduced NADH supply from TCA (Driouch et al., 2012). Similar results were found by Lu et al. (2015) who compared the metabolic changes of a glucoamylase-overexpressing *A. niger* strain and its wild-type grown on ^13^C-labelled glucose. In this case, the PPP activation might have been induced in response to a higher ATP pool, which would have inhibited the glucose-6-phosphate isomerase from glycolysis (Lu et al., 2015). This group also assessed the metabolic alterations of the same recombinant strain triggered by a sharp shift from an aerobic to oxygen-limited condition by using metabolomics, fluxomics, and transcriptomics data (Lu et al., 2018). Under oxygen limitation, increases in the glucoamylase yield, intracellular redox levels, and excretion of polyols and organic acids (e.g., oxalic acid, mannitol, and xylitol) were observed. The pool sizes of most of the intracellular metabolites were reduced, although succinate and citrate had their levels increased. Differential expression analysis revealed the up-regulation of genes from fatty acid catabolism, glyoxylate, and 4-aminobutyrate (GABA) pathways, and the down-regulation of genes of fatty acid and ribosome biogenesis in oxygen-limited conditions. According to this study, the cells would cope with the intracellular redox balance by activating the glyoxylate shunt, reducing the NADH formation through TCA, while producing the glucoamylase due to higher availability of protein precursors (Lu et al., 2018).

Given the importance of metabolomics for a better understanding of cellular metabolism, we proposed here to evaluate the intracellular metabolic profile of *T. reesei* and *A. niger* grown on glucose, lactose, carboximethylcellulose (CMC), and steam-exploded sugarcane bagasse (SEB) by proton nuclear magnetic resonance (^1^H NMR) analysis. Sugarcane bagasse is a very abundant lignocellulosic residue from Brazilian ethanol biorefineries, and it has an enormous industrial potential to be further explored in the generation of fuels and high-value chemicals (Palladino et al., 2021). This approach was used in an attempt to provide evidence at the metabolic level of which pathways of the primary metabolism are triggered by different carbon sources, and to explore in a holistic view the transcriptomics data available in the literature.

Primary metabolism provides the building blocks of vital importance for the basic functions of the cells, including biosynthesis of biomembranes, energy production, production of extracellular enzymes and secondary metabolites. Furthermore, the primary metabolism is a key for the development of strains presenting new desired traits by genetic engineering, and, therefore, its further investigation by metabolomics is needed (Andersen, 2014; Chroumpi et al., 2020). Secondary metabolism is of extreme relevance for discovery of new bioactive molecules with diverse industrial applications (e.g. plant biostimulants, biocontrol, and clinical drugs) (Rush et al., 2021; Zeilinger et al., 2016), however they were not the focus of this study, and were not evaluated here.

As far as we know, this is the first study comparing *T. reesei* and *A. niger* metabolomes on four important carbon sources. The metabolic profiling revealed interesting patterns and intra- and interspecific differences regarding the use of these carbon sources by the two industrial fungi. Our results open new perspectives for developing new fermentative bioprocesses and/or hyperproducing strains that are more robust to carbon sources of industrial interest.

## Methods

### Microorganisms and cultivation conditions

*Trichoderma reesei* RUT-C30 (ATCC 56765) and *A. niger* N402 (ATCC 64974) strains were kindly provided by Prof. Dr. Bernard Seiboth (Technischen Universität Wien, Austria) and Prof. Dr. David Archer (University of Nottingham, UK), respectively. Initially, *T. reesei* and *A. niger* spores were streaked into solid potato dextrose agar (PDA) medium (39 g L^-1^ PDA) and incubated for 10 and 3 days at 29°C and 28°C, respectively. The spores were resuspended in a solution of 8 g L^-1^ NaCl and 0.5 mL L^-1^ Tween 80, and 1 x 10^7^ spores were inoculated into 250 mL flasks containing 30 mL of Mandels-Andreotti (MA) liquid medium (pH 5.5) (Mandels and Andreotti, 1978). MA medium was composed of 20 mL L^-1^ of 50x trace element solution (0.25 g L^-1^ FeSO_4_ • 7H_2_O, 0.085 g L^-1^ MnSO_4_ • H_2_O, 0.07 g L^-1^ ZnSO_4_ • 7H_2_O, 0.1 g L^-1^ CaCl_2_ • 2H_2_O), 250 mL L^-1^ 2x mineral solution (5.6 g L^-1^ (NH_4_)_2_SO_4_, 8.0 g L^-1^ KH_2_PO_4_, 1.2 g L^-1^ MgSO_4_ • 7H_2_O, 1.6 g L^-1^ CaCl_2_ • 2H_2_O), 500 mL L^-1^ citrate phosphate buffer (0.1 M Na_2_HPO_4_, pH 5.0 adjusted with 0.2 M citric acid), 1 mL L^-1^ 5 M urea and a predetermined concentration of carbon source (10 g L^-1^ glucose, 10 g L^-1^ lactose monohydrate, 10 g L^-1^ Na-CMC, or 5 g L^-1^ SEB). SEB was obtained after pre-treatment of sugarcane bagasse as described by Borin et al. (2017). The spores were incubated at 200 rpm for 48 h at 29°C and 28°C for *T. reesei* and *A. niger*, respectively. Cultures of *T. reesei* were exposed to white light to induce cellulase expression and production (Schmoll, 2018). All media were sterilized at 121°C for 20 min before the fungi were inoculated or transferred.

The mycelia were then separated from the medium by vacuum filtration with filter paper, washed three times with sterile water, and immediately ground in liquid nitrogen. Aliquots of 100 mg were stored at -80°C for further extraction of the intracellular metabolites. The supernatants were kept at -20°C for sugar analysis, total protein quantification, and enzyme activity measurements. In order to collect the fungal biomass dry weight, another independent experiment was conducted in the same way. The mycelia grown after 48 h were collected by filtration, washed thoroughly with sterile water, dried at 80°C for three days, and then weighed using an analytical scale. Mycelia dry weight was obtained only for fungi grown on glucose, CMC, and lactose, as they were the only carbon sources soluble in water.

As it was not possible to separate the mycelium from the bagasse in SEB cultivation, aliquots of 120 mg containing both mycelium and residual SEB were harvested. This amount was chosen as the most appropriate for the identification of metabolites according to previous tests (data not shown). In addition, 100 μL aliquots of the glucose, CMC, and lactose-containing liquid media without fungal inoculum, and 120 mg aliquots of sole SEB without fungal inoculum were also stored at -80°C for further extraction of the metabolites to exclude a possible cross-identification of metabolites found in both the broth media and fungal intracellular space. These aliquots without fungi were used as blank samples for excluding false-positive metabolites, and detecting the metabolites originating uniquely from the fungi. The experiment was performed with four biological replicates.

### Extraction of metabolites

For extraction of the polar intracellular metabolites, 600 μL of a solution containing methanol (Sigma-Aldrich, USA) and chloroform (J.T.Baker, Thermo Fisher Scientific, USA) (2:1, v/v) were added to the frozen samples, vortexed for 10 s, sonicated for 5 min in an ultrasonic bath (Branson, EUA), and kept on ice for 15 min. Then 300 μL chloroform and 300 μL cold Milli-Q® water (Sigma-Aldrich, USA) were added to form a triphasic solution. Samples were vortexed for 10 s and centrifuged at 14,000 rpm for 20 min at 4°C. From the upper methanol fraction, 300 μL were lyophilized in a refrigerated concentrator (Refrigerated CentriVap Centrifugal Concentrator, Labconco, USA) at 4°C for at least 48 h for complete removal of residual methanol. Samples were resuspended in 540 μL of deuterated water (D_2_O) (Sigma-Aldrich, USA) and 60 μL of sodium phosphate buffer (1 M Na_2_HPO_4_/NaH_2_PO_4_ in D_2_O and 0.5 mM trimethylsilylpropionate (TMSP), pH 7.4). After a quick vortex and spin, the entire sample volume was transferred to NMR cylindrical transparent tubes. TMSP was used as an internal control of the experiment.

### Data processing and analysis

The ^1^H NMR spectra of the metabolites were obtained after injecting the samples into a Varian-Agilent Inova spectrometer (Agilent Technologies Inc.™, Santa Clara, USA) available at the metabolomics facility of the Brazilian Biosciences National Laboratory (LNBio-CNPEM, Campinas, Brazil). The frequency of 500 MHz ^1^H and constant temperature of 25°C were set for the analysis using a triple resonance cryogenic probe. Each sample was scanned 256 times, each having 32,000 points and an acquisition time of 4 s. A spectral width window of 16 ppm was used, and a waiting time of 1.5 s was added between the scans. During the scans, a continuous field of water pre-saturation radiofrequency was used. Spectral phase analysis, baseline correction, identification and quantification of the metabolites were performed with Chenomx NMR Suite^®^ software and Chenomx Reference Compounds database (Chenomx Inc.™, Edmonton, Canada), and confirmed by manual curation.

Normalization and statistical analyses were performed with the MetaboAnalyst 5.0 online tool (Pang et al., 2021b), using the molar concentration (μM) of the metabolites and the fresh weight (mg) of samples as data input. By default, metabolites having only missing values or a unique value across all tested conditions were excluded. Afterwards, missing values were replaced by 1/5 of the minimal positive value found for the corresponding metabolite considering all analysed conditions. Normalization was performed by dividing each molar concentration by the corresponding mass of the biological sample before converting the values into log_10_. As citrate was included in the broth medium, which could interfere with citrate from TCA cycle, it was removed from the analyses. The original, processed, and normalized data of *T. reesei* and *A. niger* grown on glucose, CMC, and lactose are available in **Table S1**, whereas data from samples grown on SEB are in **Table S2**. Metabolites identified in the culture media of glucose, CMC, lactose, and SEB without fungal inoculum are presented in **Table S3**.

Principal component analysis (PCA) and hierarchical clustering (using the Euclidean distance and the Ward clustering algorithm) were conducted to examine metabolite profile similarity among samples. Pearson’s correlation analysis was used to calculate the correlation and reproducibility of the replicates. Only metabolites having Pearson correlation > 0.7 and false discovery rate (FDR) < 0.1 were considered for analysing pattern of change through the ‘pattern search’ option in MetaboAnalyst. Moreover, metabolites having fold change ≥ 2 (log_2_ ≥ 1.0) and FDR ≤ 0.1 (equal variance) were used to identify significant differences between the fungi for the same growth condition by volcano plot.

For evaluating the putative connections between the metabolites identified in this study and the levels of transcripts associated with enzymes catalyzing their metabolic reactions, we used the studies of Bischof et al., (2013); Ries et al., (2013); Dos Santos Castro et al., (2014); dos Santos Castro et al., (2016); Borin et al., (2017a) for *T. reesei.* For *A. niger*, the studies of Delmas et al., (2012); Pullan et al., (2014); van Munster et al., (2014); Borin et al., (2017); Gruben et al., (2017) and the available transcriptomic data of Prof. Dr. Marcel Gutiérrez-Correa (Universidad Nacional Agraria La Molina, Peru) (2016) were used. Accession number of transcriptomics data, growth conditions, and fungal strains of the aforementioned studies are available in **Table S4**. The annotation of *T. reesei* RUT-C30 and *A. niger* N402 strains was used for gene identification when other fungal strain was not specified.

### Quantification of total protein

Quantification of total protein in the supernatant was measured with the fluorescent Qubit Protein Assay Kit (detection range: 0.25-5 µg) (Thermo Fisher Scientific, USA) in the Qubit 4 Fluorometer (Thermo Fisher Scientific, USA). Briefly, the concentrated dye was diluted in buffer at a ratio of 1:200, which was used for diluting the standard curve (0, 200, and 400 ng μL^-1^) and the samples to a final volume of 200 μL.

### Enzymatic activity

Enzymatic activities were measured by the 3,5-dinitrosalicylic acid (DNS) method (Miller, 1959), in which 50 μL of the substrate and 20 μL of the supernatant of each fungus were diluted in 100 mM sodium acetate buffer (pH 4.8) to a final volume of 100 μL. According to the absorbance measured, the sample was further diluted 10 times in the same buffer and the assay was repeated. The reaction was incubated at 50°C in a thermocycler for 30 min for CMC (10 g L^-1^ stock concentration), and 10 min for xylan from beechwood (5 g L^-1^ stock concentration) (Sigma-Aldrich, USA). The reaction was stopped with 100 μL of DNS at 99°C for 10 min. Absorbance was detected at 540 nm in a plate reader (Tecan Infinite M200 PRO, Switzerland), and serial dilutions of glucose and xylose were used to construct the standard curve of cellulase and xylanase activities, respectively. Each unit (IU) of enzymatic activity was defined as the amount of enzyme required to release 1 μmol of reducing sugar per min.

For the specific activities of cellobiohydrolase (CBH) and β-glucosidase (BGL), the synthetic substrates 4-nitrophenyl β-D-cellobioside (pNPC) and 4-nitrophenyl β-D-glucopyranoside (pNPG) (Sigma Aldrich, USA) were used, respectively. Activity was measured by diluting 30 μL of the supernatant in 20 μL of 100 mM sodium citrate buffer (pH 4.8) and 50 μL of the respective 4-nitrophenol (pNP) substrate (10 mM). The reaction was incubated at 50°C for 10 min for both substrates and stopped by adding 100 μL of 1 M sodium carbonate. Absorbance was detected at 410 nm and the serial pNP dilutions were used to construct the standard curve. Each unit (IU) of enzymatic activity was defined as the amount of enzyme required to release 1 μmol pNP per min. As control, enzyme-containing supernatants were added after stopping the reactions with DNS or sodium carbonate, and the detected absorbance values were subtracted from the measured activities.

### High-performance liquid chromatography (HPLC) analyses

Quantification of mono and disaccharides present in the extracellular supernatant samples was carried out by HPLC. Before injecting the samples, all supernatants were homogenized, centrifuged, and filtered through a 0.22 µm syringe PVDF-filter (Millex^®^, Sigma-Aldrich, USA). Analyses of glucose, xylose, and cellobiose were conducted at an Agilent 1260 Infinity series HPLC equipment (Santa Clara, USA) equipped with a refractive index detector at 35°C, analytical column Aminex HPX-87H (300 mm x 7.8 mm, Bio-Rad, USA) and pre-column (30 mm x 4.6 mm) at 35°C. An isocratic flow of 5 mM sulfuric acid at a flow rate of 0.6 mL min^-1^ was used for the elution of the mobile phase with an injection time of 30 min. For galactose and lactose sugars, samples were injected in an UltiMate 3000 HPLC system (Thermo Fisher Scientific Inc., USA) equipped with a refractive index detector at 55°C, analytical column Aminex HPX-87P (300 mm x 7.8 mm, Bio-Rad, US) and pre-column (30 mm x 4.6 mm) at 55°C. Milli-Q^®^ water (Sigma-Aldrich, US) was used as elution solvent at a flow rate of 0.5 mL min^-1^ and the injection time was set to 30 min. The quantification of sugars was carried out based on external calibration using standards of analytical grade. Significant differences were evaluated by a one-way analysis of variance (ANOVA) test (assuming equal standard deviation) followed by the Tukey test (adjusted P-value < 0.05) using GraphPad Prism v.8.0.2 software (San Diego, USA).

## Results and discussion

### Comparison of the primary metabolic profile of *T. reesei* and *A. niger*

**Figure 1:**
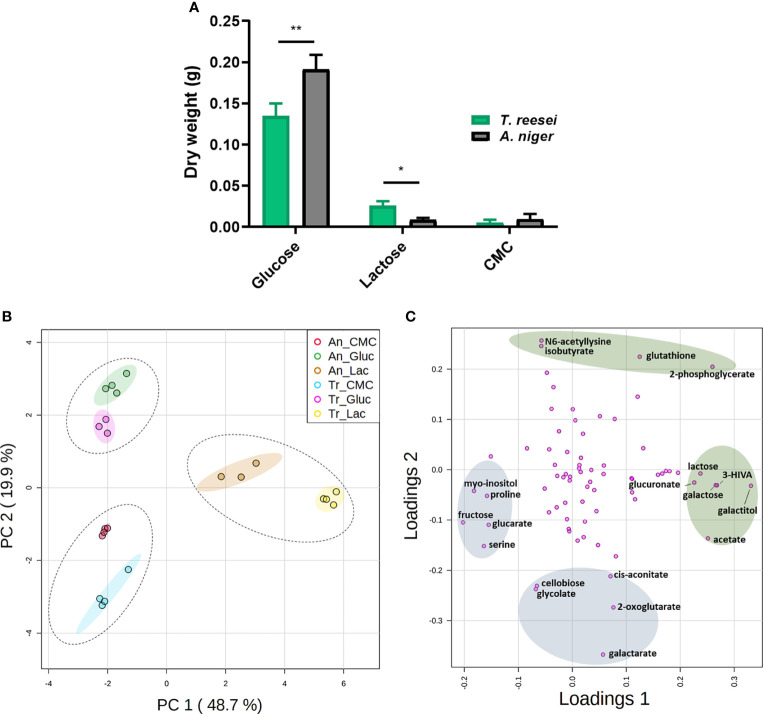
Mycelial growth and PCA analysis of the metabolome of *T. reesei* (Tr) and *A. niger* (An) grown on glucose (Gluc), lactose (Lac), and CMC for 48 h. **(A)** Dry weight of mycelia. The statistical difference between both fungi for the same condition was determined by the two-tailed unpaired Student t-test (P-value: * < 0.05; ** < 0.01). **(B)** Principal components 1 and 2 (68.6% of total variance) of fungi grown on glucose, CMC, and lactose. Dotted and solid ellipses show the three large groups formed and regions with more than 95% of reliability, respectively. **(C)** PCA loading plot of the metabolites responsible for the observed variation in *T. reesei* and *A. niger*. Only metabolites with the highest scores are named. The metabolites highlighted in green and blue show the highest and lowest score values for the first and second components, respectively. 3-HIVA, 3-hydroxyisovalerate.

Strains of *T. reesei* and *A. niger* were grown on glucose, CMC, and lactose for 48 h before having their mycelia collected to examine their growth (**Figure 1A**). In this analysis, SEB samples were not taken into consideration as the fungal biomass cannot be separated from the bagasse matrix. Comparing both fungi, *A. niger* had significantly more biomass in glucose, and *T. reesei* grew more in lactose (**Figure 1A**).

Glucose is the energy molecule preferred by filamentous fungi (Druzhinina and Kubicek, 2017a) and it is not surprising that both fungi grew more in this sugar (**Figure 1A**), which was depleted after 48 h (**Figure S1**). Lactose is a disaccharide formed by galactose and glucose, and it is well reported in the literature that lactose and galactose are not the preferred carbon sources of *A. niger* (Hasija and Wolf, 1969; Fekete et al., 2012; de Vries et al., 2017). Conversely, *T. reesei* has a higher growth rate in galactose (Fekete et al., 2012), but in our experiment, no significant difference was observed for this sugar in the supernatant of both fungi (**Figure S1**). This metabolic ability might favor the lactose catabolism to generate energy and, consequently, promote the greater growth of *T. reesei* (**Figure 1A**). Furthermore, growth delay by lactose has also been observed previously for yeast species and the excess of lactose uptake is believed to be cytotoxic for the cell as it increases the intracellular osmotic pressure (Lodi and Donnini, 2005; de Ruijter et al., 2020).

In CMC, a low amount of mycelia was observed for both fungi with no significant difference between them (**Figure 1A**), most likely due to the recalcitrant cellulosic structure not readily assimilable by the fungi (Mattam et al., 2022). However, the disaccharide cellobiose, derived from cellulose hydrolysis, was found exclusively in the supernatant of *T. reesei* (**Figure S1**). It remains elusive whether cellobiose was produced and totally consumed by *A. niger*, or whether the cellobiose consumption/production ratio was lower for *T. reesei.*

^1^H NMR-based metabolomic analysis was then performed to explore the changes in the primary metabolism of these microorganisms in different growth conditions. First, the metabolites of broth media of each carbon source without fungal inoculum were extracted and evaluated as negative controls. Only 11 metabolites were found, and among them, glucose and lactose were the most representative (> 2000 µM) in their respective carbon sources (**Table S3**). These concentrations of glucose and lactose in the media were several orders of magnitude higher than the other few metabolites found in the same or different carbon sources. This striking difference is expected as glucose and lactose were the only carbon sources added into the media (except for citrate) and are water-soluble sugars, whose structure is much less complex than CMC or SEB.

In the intracellular metabolome of *T. reesei* and *A. niger* we identified a total of 74 compounds, namely 12 sugars and sugar alcohols, 21 organic acids, 17 amino acids, 5 nucleotides, and 19 metabolites from other classes (**Table S1**). Among them, we found intermediates from glycolysis, TCA cycle, metabolism of galactose, metabolism of glutathione and GABA, and metabolites having different roles in fungal physiology, including cell wall homeostasis, stress response, and virulence. The variation between the metabolic profiles was then assessed by PCA (**Figure 1B**), and the metabolites having the highest variation over the samples were identified by loading plot (**Figure 1C** and **Table S5**).

PCA detected two outliers (samples *T. reesei* glucose replicate 2 and *A. niger* lactose replicate 1, data not shown) with higher deviation to the other samples from the same conditions, and were removed from further analysis. Therefore, these two samples are not presented in **Table S1** and **Figure 1B**. After outlier removal, PCA simulations indicated a total variance of 68.6, 77.9, 85.6, and 89.3% explained by two, three, four, and five components, respectively. Two-component analysis revealed the presence of three large sample groups that were separated essentially by the carbon source (**Figure 1B**). Thus, the carbon source was more discriminative than the fungal species. The first component (PC1) accounted for approximately half (48.7%) of the total variance and separated the lactose-grown fungi from the other growth conditions. Together with PCA (**Figure 1B**), the Pearson correlation and the hierarchical dendrogram (**Figure S2**) showed that the metabolome of the biological samples was similar within each growth condition, and lactose samples were the most distinct from the other carbon sources.

Although *T. reesei* (Sordariomycetes) and *A. niger* (Eurotiomycetes) belong to different classes and have evolved independently over time (Schoch et al., 2009; Beimforde et al., 2014), the grouping of samples according to the carbon sources indicate similar primary metabolomic profiles and, subsequently, common responses to those growth conditions. Common molecular components in the transcriptional regulation of the (hemi)cellulolytic machinery of *T. reesei* and *A. niger* supports this hypothesis (Stricker et al., 2008).

Concerning the metabolites that most contributed to the observed differences, galactitol, 3-hydroxyisovalerate (3-HIVA), galactose, 2-phosphoglycerate, acetate, and lactose showed the highest score values in PC1; and the lowest values were found for fructose, myo-inositol, serine, proline, and glucarate (**Figure 1C** and **Table S5**). In PC2, the metabolites having the highest and lowest scores were separated according to their presence – nearly exclusive - in lactose and glucose, or in lactose and CMC conditions, respectively (**Figure 1C** and **Tables S1**, **S5**).

**Figure 2:**
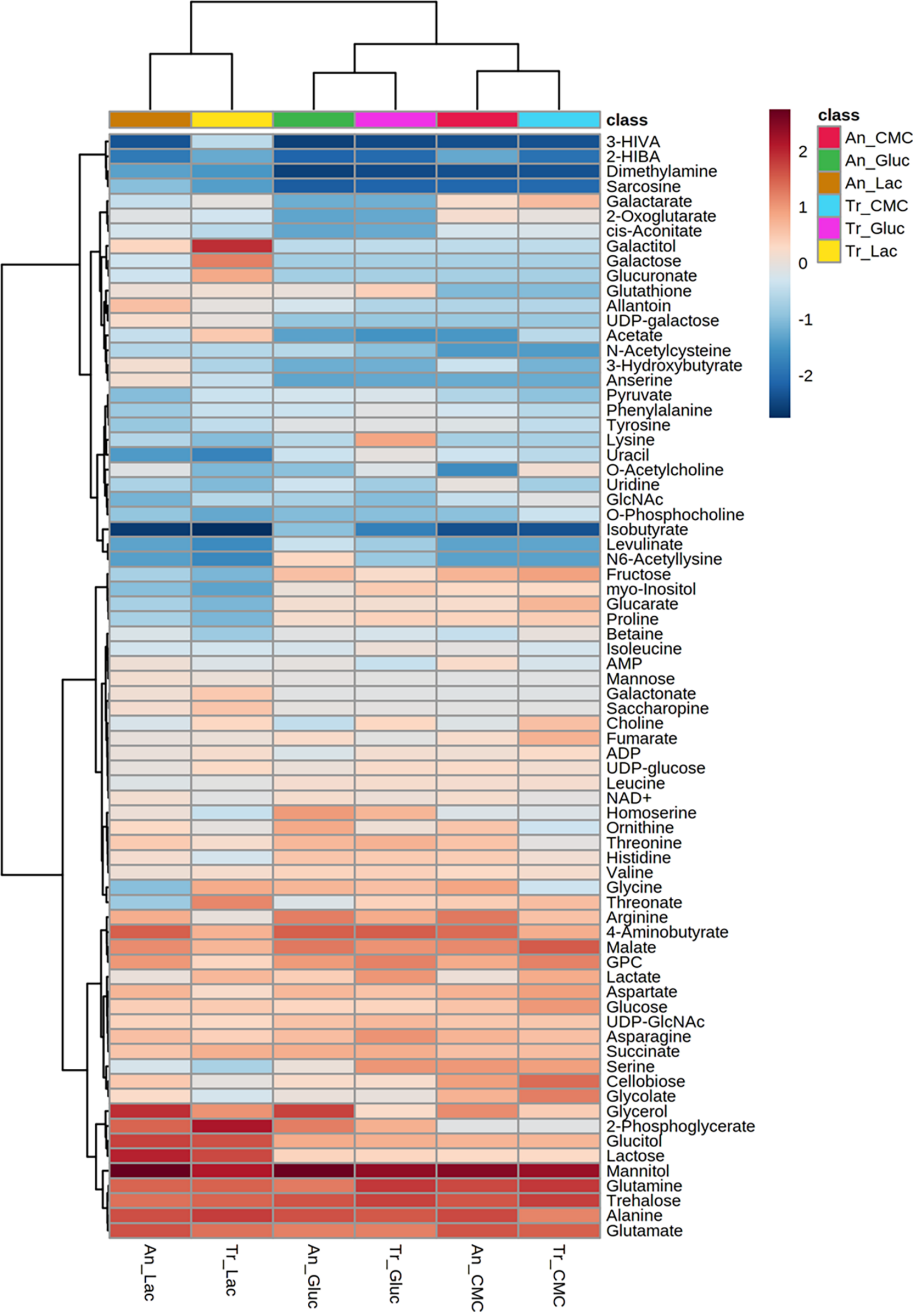
Heatmap of metabolites identified in *T. reesei* (Tr) and *A. niger* (An) grown on glucose (Gluc), CMC, and lactose (Lac) for 48 h. The abundance of each metabolite is presented as the normalized values of each condition after hierarchical clustering and scaling according to the carbon sources. 3-HIVA: 3-hydroxyisovalerate; 2-HIBA: 2-hydroxyisobutyrate; GlcNAc: N-acetylglucosamine; GPC: sn-glycero-3-phosphocholine; UDP-GlcNAc: uridine diphosphate N-acetylglucosamine.

Analysing the global concentration of metabolites in both fungi (**Figure 2**), it is possible to observe similarities in the profiles with some of them standing out from the others by the higher abundance in the conditions evaluated. Notably, the osmolytes mannitol and trehalose, and the amino acids glutamine, alanine, and glutamate were the most abundant (log_10_ > 3.0) metabolites in glucose, CMC, and lactose (**Figure 2** and **Table S1**), and were also highly abundant in SEB (**Table S2**).

Mannitol and trehalose are normally produced in fungal cells as carbohydrate and energy stock. They are extremely relevant for fungal development and act as protective molecules against various types of stress (Meena et al., 2015; Wang et al., 2018a). Mannitol is a sugar alcohol found in spores, fruiting bodies, and mycelia. It is consumed during spore germination under carbon starvation, plays a role as an osmolyte, is a quencher of reactive oxygen species (ROS), and it still might confer resistance to metal stress (Meena et al., 2015; Perdigão Cota de Almeida et al., 2021). Trehalose is a disaccharide formed by two glucose molecules connected by an α-1,1 ligation. It is found in bacteria, insects, plants, and fungi, playing roles as a storage molecule and protector against abiotic stress. It might be used during glycolysis, sporulation, or conidial germination. Furthermore, trehalose helps the cell withstand stresses caused by nutrient limitation in the medium, prevents the aggregation of denatured proteins, and participates in cell wall homeostasis (Al-Bader et al., 2010; Thammahong et al., 2019). For these reasons, it is evident that mannitol and trehalose are of paramount importance for the growth and adaptation of both fungi in all four carbon sources analysed.

Concerning the amino acids, it is known that the citrate and ammonium (NH_4_^+^) available from the media might be uptaken by the cells and converted into glutamate (Ząbek et al., 2017; Tang et al., 2020). Glutamate participates in the leucine (Steyer et al., 2021) and 2-oxoglutarate synthesis (Pang et al., 2021a), and it can be reversibly converted into glutamine, another amino acid necessary for protein biosynthesis and used by filamentous fungi as one of the most preferred sources of nitrogen alongside NH_4_^+^ (Pang et al., 2021a). Glutamine has a prevailing role in fungal physiology as it might trigger the target of rapamycin (TOR) kinase pathway responsible for regulating diverse processes, including vegetative growth, stress response, and cellulase induction (Lv et al., 2015; Ries et al., 2018a). Additionally, glutamine is considered the main signal molecule activating nitrogen catabolite repression (NCR) of cellulase synthesis, similarly to glucose and carbon catabolite repression (Tudzynski, 2014; Pang et al., 2021a).

Alanine was also detected in higher levels across all samples analysed (**Figure 2** and **Tables S1**, **S2**). Alanine is naturally very common in the amino acid composition of mycelia (Watkinson, 2016) and it is the main precursor of gluconeogenesis (Karimi et al., 2019). By a transamination reaction, alanine is converted reversibly into pyruvate, which in turn might be used as a substrate in reactions of gluconeogenesis, protein, and fatty acid synthesis. The alanine backbone is still believed to be incorporated into the formation of stable cell components, such as cell walls and membranes (Gunina et al., 2014). Finally, the importance of alanine in the microbial central metabolism is not limited to its role as a nitrogen and carbon source, but also by participating in cell response against salt stress (Ding et al., 2019), acting like a nitrogen sink for ammonium accumulation (Kerkaert et al., 2022), and even being an active player in the coordination of bacterial biofilms (Díaz-Pascual et al., 2021).

Glutamine, glutamate, and alanine have also been previously reported in higher concentrations in the *T. atroviride* conidia (Polozsányi et al., 2021), in the fungal pathogen *Geotrichum candidum* grown on potato dextrose broth (Ząbek et al., 2017), and in *Neurospora crassa* when transferred from sucrose to acetate-containing media (Thomas and Baxter, 1987). Additionally, alanine and glutamine were the highest detectable amino acids in the biomass of the fungi *A. oryzae, N. intermedia*, and *Rizhopus oryzae* grown on vinasse from ethanol refinery (Karimi et al., 2019). Even actinomycetes isolated from the rhizosphere have shown increased intracellular pools of glutamate and alanine (Chalot and Brun, 1998). The identification of these amino acids in our study and in other works using different fungi and growth conditions reinforces their role as determinant amino acids of distinct metabolic pathways.

GABA, glycerol-3-phosphocholine, glycerol, and malate have also drawn attention for their high levels in *T. reesei* and *A. niger* grown on all different carbon sources (**Figure 2** and **Tables S1**, **S2**). GABA is a non-protein amino acid formed from glutamate decarboxylation and with a central role in the GABA shunt pathway that deflects the TCA cycle carbon flux to form succinic semialdehyde and succinate. This pathway is less energy efficient than the TCA cycle as it does not generate an ATP or NADH gain for the cell, and it has a role not yet fully understood in fungal metabolism (Mead et al., 2013). In addition to the GABA shunt pathway, GABA is also produced in response to various stresses and under limited carbon source availability (Bönnighausen et al., 2015; Shelp et al., 2017). It acts on conidiospore germination and can serve as a source of nitrogen for fungi (Kumar and Punekar, 1997). Still, GABA is a valuable molecule precursor of feedstocks used in the plastic industry for manufacturing nylon 4 (Park et al., 2020).

Glycerol and glycerol-3-phosphocholine are involved in the metabolism of phospholipids that compose the cell membranes. The former binds a phosphate molecule to two fatty acid chains and it works as an energy molecule and also plays a stress protective role (Klein et al., 2017; Ries et al., 2018b). The latter participates in the elongation of the lipid chains by donating methyl groups along with its degradation product choline (Ząbek et al., 2017).

Malate is a C4-carboxylic acid and key intermediate in the TCA cycle. It might be either formed by the oxidative and reductive part of the TCA cycle, or by the glyoxylate shunt pathway through the conversion of fumarate, oxaloacetate, and glyoxylate, respectively (Gu et al., 2018; Chroumpi et al., 2020). Malate accumulation in the cells is believed to be due to the malate found in the cytosol and produced from the reductive TCA cycle (Chroumpi et al., 2020). In the cytosol, malate can be converted into pyruvate, NADH, and CO_2_ by the malic enzyme, whose function is essential for fatty acid biosynthesis (Ratledge, 2014; Passoth, 2017). Taken together, the high levels of GABA, glycerol, glycerol-3-phosphocholine, and malate in both fungi and all conditions demonstrate metabolic changes towards stress and nitrogen metabolism (GABA), biosynthesis of lipids and membranes (glycerol and glycerol-3-phosphocholine), and generation of energy (malate).

### Primary metabolic profile by carbon source

As the carbon sources contributed to the largest variation among samples, they were individually analysed to identify which pathways were modulated upon each carbon source in *T. reesei* and *A. niger*. Comparative analysis of metabolic profiles provided clues of how both fungi used these substrates not only to grow but also to produce enzymes of industrial interest, such as cellulases and hemicellulases, as indicated by the enzymatic activities assayed in this study.

#### Glucose

**Figure 3:**
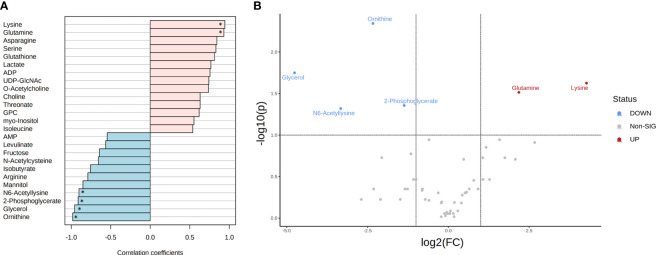
Metabolite correlation and significant differences in the metabolome of *T. reesei* and *A. niger* grown on glucose for 48 h. **(A)** List of the top 25 metabolites with higher correlation patterns in *T. reesei* (pink) and *A. niger* (blue). Metabolites having Pearson correlation > 0.7 and FDR < 0.1 are marked (*). **(B)** Volcano plot of metabolites with significant difference between both fungi. Metabolite levels were increased (red) or reduced (blue) in *T. reesei* compared to *A. niger*. ADP, adenosine diphosphate; UDP-GlcNAc, uridine diphosphate N-acetylglucosamine; GPC, sn-glycero-3-phosphocholine; AMP, adenosine monophosphate.

In total, 49 and 46 metabolites (detected in at least three replicates) were identified for both *T. reesei* and *A. niger* grown on glucose (**Table S1**). To further investigate which compounds had concentration changes that followed a particular pattern, i.e. were more significantly abundant in one species in that specific substrate, we performed a correlation analysis (**Figure 3A**). Lysine and glutamine were the metabolites with higher correlation (Pearson correlation > 0.7, FDR < 0.1) in *T. reesei* (more abundant in *T. reesei* than in *A. niger)*, while ornithine, glycerol, 2-phosphoglycerate, and N6-acetyllysine showed the opposite effect (more abundant in *A. niger* than in *T. reesei*). Coincidently, these were the only metabolites with significant differences (fold change (FC) > 2, FDR < 0.1) between both fungi (**Figure 3B** and **Table S6**).

**Figure 4:**
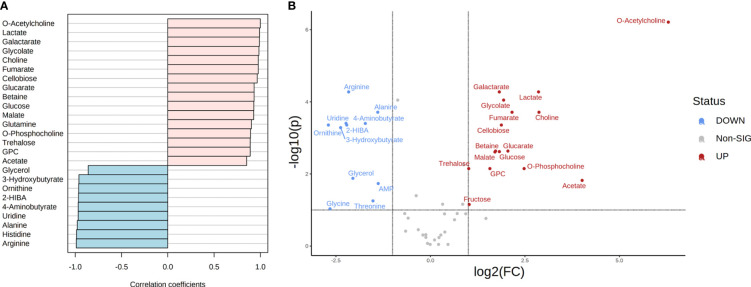
Metabolite correlation and significant differences in the metabolome of *T. reesei* and *A. niger* grown on CMC for 48 h. **(A)** List of the top 25 metabolites with higher correlation patterns in *T. reesei* (pink) and *A. niger* (blue). All metabolite correlation coefficients shown were significant (Pearson correlation > 0.7 and FDR < 0.1). **(B)** Volcano plot of metabolites with significant difference between both fungi. Metabolite levels were increased (red) or reduced (blue) in *T. reesei* compared to *A. niger*. 2-HIBA, 2-hydroxyisobutyrate; AMP, adenosine monophosphate; GPC, sn-glycero-3-phosphocholine.

Regarding the metabolites differentially altered in *A. niger*, glycerol was considerably more abundant (log_2_(FC) = -4.76) compared to *T. reesei* grown on glucose (**Figure 3B** and **Table S6**). Curiously, this metabolite was found to be significantly higher in all growth conditions in *A. niger* compared to *T. reesei* (**Figures 4****–** **6** and **Table S6**). Apart from being an important structural element of the cell membranes and lipid storage, glycerol also plays a role as a protective agent against osmotic and temperature stress (Wyatt et al., 2015; Klein et al., 2017; Ries et al., 2018b). Glycerol accumulation by *A. niger* after 48 h of growth on glucose might be a strategy to avoid water loss and surpass the hypertonic milieu (Kalampokis et al., 2020).

Ornithine and 2-phosphoglycerate were also significantly changed in *A. niger* compared to the glucose-grown *T. reesei* (**Figure 3** and **Table S6**). Ornithine is part of the urea cycle and the molecule precursor of siderophore biosynthesis. It is formed from the arginine and glutamate degradation in the cytosol and mitochondria, respectively (Were et al., 2022), and also participates in polyamine metabolism, giving rise to putrescine, spermidine, and spermine. Polyamines are involved in the modulation of several intracellular processes, such as cell growth, morphogenesis, signal transduction, and oxidative stress response (Rocha and Wilson, 2019). One hypothesis to explain why none of these three polyamines has been identified in our metabolomics data is that ornithine might be consumed within the urea cycle to form intermediates in other pathways such as fumarate and arginine. Although arginine did not accumulate in *A. niger* grown on glucose, it was correlated with this carbon source (Pearson correlation 0.789, FDR 1.68E-01, **Table S6**) and more abundant in *A. niger* grown on CMC and lactose compared to *T. reesei* (**Table S6**), supporting the proposed hypothesis.

2-phosphoglycerate is formed as one of the intermediates of the glycolytic and gluconeogenic pathways, and it was also elevated in *A. niger* grown on glucose compared to *T. reesei* (**Figure 3B** and **Table S6**). As a readily assimilable sugar, glucose should favor carbon catabolism, and, therefore, the glycolysis pathway. Hence, glycolysis might be activated for converting glucose into pyruvate to feed the oxidative TCA cycle. At the same time, glycolysis might provide intermediates that are precursors of a diverse set of macromolecules needed for fungal growth, including amino acids and nucleotides. *Aspergillus niger* had higher growth in glucose than *T. reesei* (**Figure 1A**), confirming its high growth rate and energy demand.

#### CMC

In the presence of this carbon source, 19 and 12 metabolites showed a higher correlation in *T. reesei* and *A. niger*, respectively (**Figure 4A** and **Table S6**). All metabolites found with significant difference between both fungi were also correlated to their respective species, except glycine which was more abundant in *A. niger* (log₂ (FC) = -2.66) but was not correlated to this fungus (Pearson correlation -0.693, FDR 9.24E-02) (**Figure 4** and **Table S6**). For *T. reesei*, significant differences were observed for metabolites of glycerophospholipid metabolism (choline, O-acetylcholine, O-phosphocholine, and glycerol-3-phosphocholine), pyruvate metabolism (lactate), ascorbate metabolism (galactarate and glucarate), TCA cycle and glyoxylate shunt (malate, fumarate, and glycolate), as well as glucose and the disaccharides cellobiose and trehalose (**Figure 4B** and **Table S6**).

Phosphatidylcholine is the main glycerophospholipid found in eukaryotic cell membranes and comprises 68% and 51% of the *T. reesei* and *A. niger* membrane phospholipids, respectively (Gryz et al., 2019). The accumulation of choline, O-acetylcholine, O-phosphocholine, and glycerol-3-phosphocholine suggests that *T. reesei* is shifting part of its metabolic response to the formation and/or remodeling of the cell membrane (Gow et al., 2017). Alternatively, *T. reesei* might mobilize the pool of these metabolites, especially glycerol-3-phosphocholine, towards the β-oxidation of fatty acids to provide more acetyl-CoA and fuel the TCA cycle, for example. Meanwhile, further lipidomic analysis of both fungi is necessary to reveal the metabolic alterations related to fatty acid metabolism.

**Figure 5:**
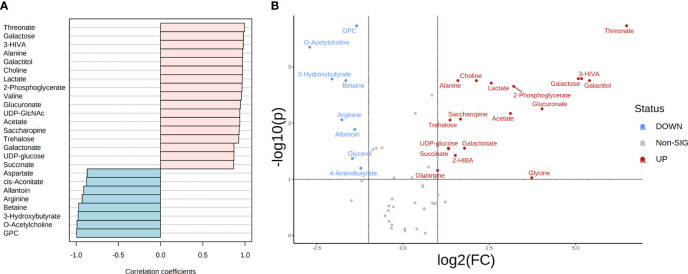
Metabolite correlation and significant differences in the metabolome of *T. reesei* and *A. niger* grown on lactose for 48 h. **(A)** List of the top 25 metabolites with higher correlation patterns in *T. reesei* (pink) and *A. niger* (blue). All metabolite correlation coefficients shown were significant (Pearson correlation > 0.7 and FDR < 0.1). **(B)** Volcano plot of metabolites with significant difference between both fungi. Metabolite levels were increased (red) or reduced (blue) in *T. reesei* compared to *A. niger*. 3-HIVA, 3-hydroxyisovalerate; UDP-GlcNAc, UDP-GlcNAc - uridine diphosphate N-acetylglucosamine; GPC, sn-glycero-3-phosphocholine; 2-HIBA, 2-hydroxyisobutyrate.

Interestingly, lactate was significantly more abundant in *T. reesei* grown on CMC (**Figure 4** and **Table S6**), lactose (**Figure 5** and **Table S6**), and SEB (**Table S2**). The conversion of pyruvate to lactate is catalyzed by the enzyme lactate dehydrogenase, in a reaction usually performed by anaerobic lactic acid bacteria, or aerobically by the filamentous fungi of the genus *Rhizopus* sp. (Juturu and Wu, 2016; Komesu et al., 2017). This reaction oxidizes the NADH to NAD^+^, which can be used to convert glyceraldehyde-3-phosphate to 1,3-bisphosphoglycerate in the glycolytic pathway. Although *T. reesei* and *A. niger* are not considered natural lactate producers and perform aerobic respiration, it is possible to identify putative genes encoding lactate dehydrogenase in the genome of both fungi. According to the KEGG database, only *A. niger* has a predicted lactate dehydrogenase (An04g08220, EC 1.1.1.27). However, there are more six and at least nine genes having putative lactate dehydrogenase activity annotated for *A. niger* and *T. reesei* according to Andersen et al., (2008) and the JGI database, respectively (**Figure S3**). The characterization of those genes is necessary to evaluate their function and relationship with lactate formation. This is interesting because lactate is also a building block for the production of poly(lactic acid) and poly(lactic-co-glycolic acid), the two biodegradable plastics most used nowadays (Djukić-Vuković et al., 2019). And curiously, lactate was also observed in the metabolome of *A. nidulans* grown on glucose and CMC (Ries et al., 2018b). Glucose and cellobiose were more elevated in *T. reesei* grown on CMC than in *A. niger* (**Figure 4** and **Table S6**). This might be due to the hypercellulolytic profile of *T. reesei* characterized by the great production of extracellular cellobiohydrolases, for example, which convert cellulose into cellobiose. However, growth in CMC was similar for both fungi (**Figure 1A**).

In *A. niger*, the major metabolites with significant differences participate in pyrimidine metabolism (uridine), ketone body degradation (3-hydroxybutyrate and 2-hydroxyisobutyrate), urea cycle (ornithine), amino acid metabolism (arginine, alanine, threonine, glycine), and stress response (glycerol and GABA) (**Figure 4** and **Table S6**).

In CMC, 3-hydroxybutyrate and 2-hydroxyisobutyrate were found in trace amounts in *A. niger* only (**Table S1**). These metabolites can be produced from acetyl-CoA through various intermediate reactions and especially 2-hydroxyisobutyrate has drawn increasing attention from pharmaceutical and chemical industries aiming to use it as a precursor molecule for various high-added value products, such as biodegradable polyhydroxybutyrate plastics (Rohwerder and Müller, 2010; Raberg et al., 2018).

The significant presence of amino acids glycine, arginine, threonine, and alanine in the metabolome of *A. niger* grown on CMC was evident (**Figure 4** and **Table S6**). Amino acid metabolism is a complex network full of interconnected biochemical pathways that lead to the formation of several intermediates. Alanine, for example, may originate or be formed from pyruvate via an aminotransferase. Arginine, when cleaved by arginase, is converted into ornithine upon release of urea. Ornithine serves as a precursor to polyamine biosynthesis, and threonine to glycine biosynthesis (Kanehisa et al., 2021; Kerkaert et al., 2022). The accumulation of these amino acids in *A. niger* seems to be important for the fungal growth and adaptation in this carbon source.

#### Lactose

Lactose was the main carbon source responsible for differences in the metabolic profile of *T. reesei* and *A. niger* (**Figures 1B, C**). In total, 59 and 44 metabolites (in at least three replicates) were found for *T. reesei* and *A. niger*, respectively. As mentioned earlier, lactose is a slow metabolizing sugar for both fungi, but it affects more severely *A. niger* growth, impairing conidiospore germination and mycelium formation (Horr, 1936; Karaffa et al., 2006). This difference in the metabolism is reflected directly in the higher number of metabolites showing greater correlation (**Figure 5A**) and accumulation in *T. reesei* (**Figure 5B**). Among them, threonate, glucuronate, galactose, galactitol, and 3-HIVA were found to be relevant (**Figure 5** and **Table S6**). Regarding the *A. niger* metabolome, O-acetylcholine, 3-hydroxybutyrate, arginine, and betaine showed higher correlation patterns and were elevated when this species was grown on lactose (**Figure 5** and **Table S6**).

Threonate is the degradation product of ascorbic acid, and it was found exclusively in *T. reesei* grown on lactose (**Figure 5** and **Table S1**). Ascorbic acid is an antioxidant molecule involved in the detoxification of ROS, maintenance of the activity of iron-dependent enzymes, and uptake of iron (Smirnoff, 2018). Although ascorbic acid was not detected in the metabolome of both fungi (nor its analogue D-erythroascorbate) (Smirnoff, 2018), which was observed in the basidiomycete *Phanerochaete chrysosporium* (Miura et al., 2004), there is evidence that fungi and yeast can produce ascorbic acid (Porro and Sauer, 2000; Makut and Ogiri, 2017; Banjo et al., 2019). Even more intriguing, ascorbate biosynthesis relies on intermediates from galactose metabolism in plants, glucuronate in mammals, and galactonate in photosynthetic protists (Smirnoff, 2018), metabolites more abundant when lactose was the carbon source (**Tables S1**, **S6**). Thus, threonate and glucuronate may indicate that *T. reesei* cells utilize ascorbate or some analogue molecule to circumvent possible detrimental effects of oxidative stress caused by, for example, the galactose released from lactose. Galactose accumulation is reported to be toxic even at low concentrations for filamentous fungi (Horr, 1936; Karaffa et al., 2006).

Galactose and galactitol were also significantly elevated in *T. reesei* than *A. niger* when grown on lactose (**Figure 5** and **Table S6**). Although both fungi have the major galactose catabolism pathways which convert galactose into galactose-1-phosphate (Leloir pathway) or galactitol (oxidoreductive pathway) (Seiboth et al., 2007; Mojzita et al., 2012), *T. reesei* has better ability to grow on galactose (Fekete et al., 2012), as shown by its higher biomass on this substrate (**Figure 1A**).

Lactose was detected in the metabolome of *T. reesei* and *A. niger* (**Figure 1** and **Table S1**) and also in the extracellular supernatant (**Figure S1**), albeit no significant difference was found between both species. The assimilation and further catabolism of lactose in *T. reesei* is reported to be mediated by transporters anchored in the fungal surface and whose function might be also associated with the activation of its cellulolytic system (Ivanova et al., 2013; Porciuncula et al., 2013; Zhang et al., 2013; Nogueira et al., 2018; Havukainen et al., 2020). Equivalently, a few lactose, galactose and other sugar transporters have been identified for *A. niger* (Fekete et al., 2016; Peng et al., 2018; de Ruijter et al., 2020; Lin et al., 2020). Thus, our data give evidence that lactose was not only cleaved into glucose and galactose monomers as shown by the sugar quantification in the supernatant (**Figure S1**), but it was also internalized to be assimilated into the fungal energy catabolism.

3-HIVA was virtually exclusive to the *T. reesei* metabolome when grown on lactose (**Table S1**), and it was strongly correlated and elevated in this fungus (**Figure 5** and **Table S6**). 3-HIVA is one product of leucine catabolism and its accumulation seems to be toxic for the fungal cell, as *A. nidulans* mutant strains grown on leucine as the sole carbon source showed impaired growth (Rodríguez et al., 2004). However, the relevance of 3-HIVA for *T. reesei* grown on lactose remains to be further investigated.

Profiling of *A. niger* metabolome in lactose showed an accumulation of O-acetylcholine in comparison to *T. reesei* (**Figure 5** and **Table S6**). The same metabolite was also elevated in *T. reesei* grown on CMC (**Figure 4** and **Table S6**). The opposite pattern of O-acetylcholine accumulation between both fungi in CMC and lactose could not be further explored in this study, but this compound participates in the metabolism of phospholipids, and therefore it might contribute to the formation of membranes and other lipidic structures.

3-hydroxybutyrate was also increased in *A. niger* (**Figure 5** and **Table S6**). 3-hydroxybutyrate is a ketone body used in the biosynthesis of natural polymers and with ROS-scavenging properties in bacteria (Koskim et al., 2016). This metabolite was found in low concentrations in both fungi grown on lactose (**Table S1**) and was previously detected in ascomycete (Quang et al., 2003). However, according to the KEGG database, no evidence of its biosynthesis exists in *A. niger* or *T. reesei*.

**Figure 6:**
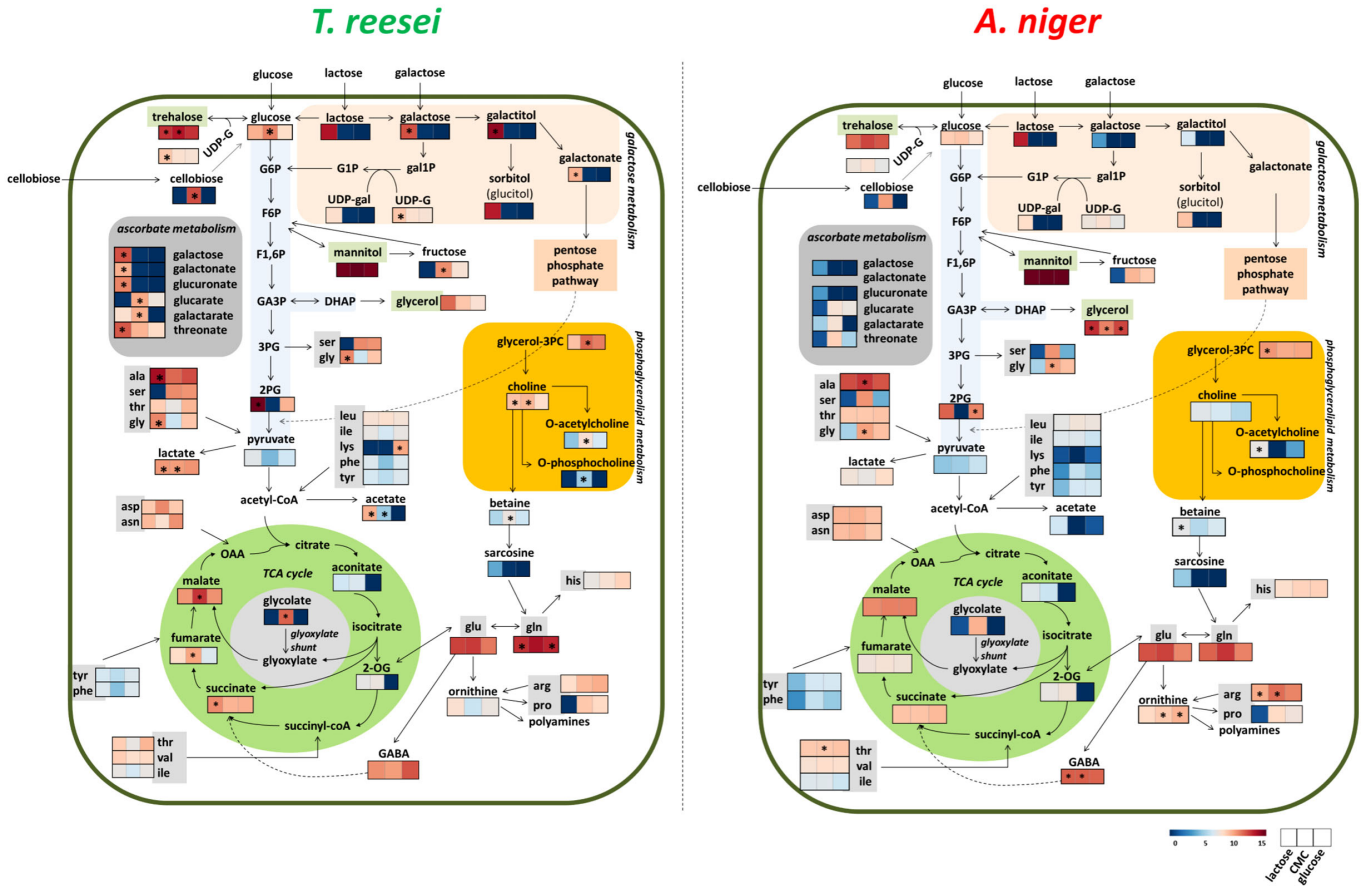
Overview of the metabolic alterations observed in *T. reesei* and *A. niger* grown on glucose, CMC, and lactose. The carbon sources in the broth media can be transported directly to the cell interior (glucose and lactose) or must be extracellularly hydrolyzed before entering the cells (CMC). For simplicity, some reactions involving intermediates that were not identified in our metabolomics analysis are not shown. Metabolites with significant differences for the same carbon source between the two fungi are highlighted (*). G1P, glucose 1-phosphate; G6P, glucose 6-phosphate; F6P, fructose 6-phosphate; F1,6P, 1,6-bisphosphate fructose; GA3P, glyceraldehyde 3-phosphate; DHAP, dihydroxyacetone phosphate; 3PG and 2PG, 3- and 2-phosphoglycerate; gal1P, galactose 1-phosphate; UDP-G, uridine diphosphate glucose; UDP-gal, uridine diphosphate galactose; TCA, tricarboxylic acid; 2-OG, 2-oxoglutarate; OAA, oxaloacetate; glycerol-3PC, glycerol 3-phosphoglycerate; ala, alanine; ser, serine; tre, threonine; gly, glycine; leu, leucine; ile, isoleucine; lys, lysine; phe, phenylalanine; tyr, tyrosine; asp, aspartate; asn, asparagine; arg, arginine; pro, proline; his, histidine; glu, glutamate; GABA, 4-aminobutyrate.

Arginine was more significantly abundant in *A. niger* than *T. reesei* in lactose (**Figure 5** and **Table S6**). This proteinaceous amino acid has been recently associated with a new biosynthetic route for the production of nitric oxide (NO) in *A. nidulans* (Franco-Cano et al., 2021). Accordingly, the mobilization of arginine from different cellular compartments allows its conversion into citrulline forming NO, which then regulates nitrogen metabolism, conidiation, and pathogenesis (Franco-Cano et al., 2021). This discovery is supported by the role of arginine as a stock of organic nitrogen (Deng et al., 2021). Therefore, arginine accumulation in *A. niger* might contribute to balancing nitrogen and carbon metabolism, or even by having a signaling function through NO. Betaine, also elevated in *A. niger* in this condition (**Figure 5** and **Table S6**), might have a synergistic effect with arginine, as it is also considered a source of carbon and nitrogen, in addition to its primary protective role against abiotic stresses (Zou et al., 2016). To facilitate a better overview and understanding of the metabolic changes found in *T. reesei* and *A. niger* grown on glucose, CMC, and lactose, a simplified scheme is presented (**Figure 6**).

#### SEB

**Figure 7:**
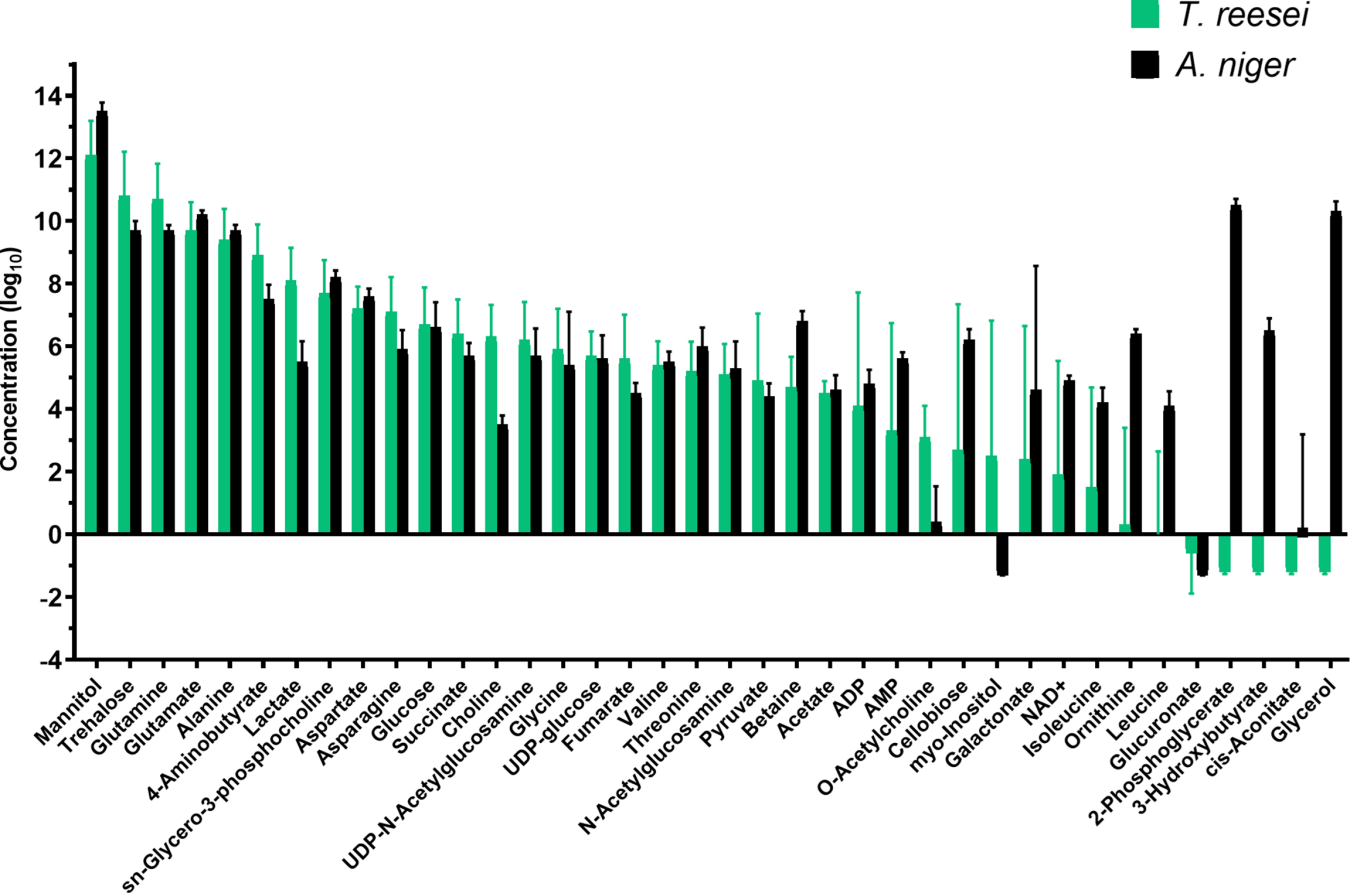
Metabolic profile of *T. reesei* and *A. niger* grown on SEB for 48 h. The pool size of each metabolite was normalized by the total mass of mycelia and SEB, and converted into log_10_ values.

In total, 26 and 35 metabolites were identified in the *T. reesei* and *A. niger* metabolome, respectively, considering the identification in at least three replicates (**Table S2**). Although it is not possible to compare the metabolome of both fungi, the most elevated metabolites were identical in both species grown on SEB (**Figure 7**). Similarly to the other carbon sources, mannitol, glutamine, trehalose, alanine, and glutamate were the most abundant in both fungi (**Figure 7**). This group of metabolites was previously discussed and seems to be of fundamental importance for the growth of *T. reesei* and *A. niger* under our evaluated conditions. Interestingly, this is also true for SEB, which is the most complex carbon source in terms of structure (crystalline and amorphous regions) and chemical composition (lignin-interspersed cellulose and hemicellulose chains, hemicellulose decorated with several different monomers, and lignin highly recalcitrant) compared with glucose, CMC, and lactose.

An evident difference was the concentration of 2-phosphoglycerate, glycerol, 3-hydroxybutyrate, ornithine, and leucine which virtually were exclusive of the *A. niger* metabolome (**Figure 7** and **Table S2**). These metabolites are part of glycolysis (2-phosphoglycerate), membrane biosynthesis and stress response (glycerol), ketone body degradation (3-hydroxybutyrate), urea cycle (ornithine), and amino acid metabolism (leucine). Notably, the presence of 2-phosphoglycerate in SEB, exclusively in *A. niger*, suggests that this fungus may be able to degrade and assimilate more easily the sugars released from the lignocellulosic biomass and convert them into intermediates of the glycolytic pathway for energy generation. Some of these sugars are inducers of the (hemi)cellulolytic arsenal of *A. niger* and *T. reesei*, enhancing the degradation rate of SEB.

### Enzymatic activity

**Figure 8:**
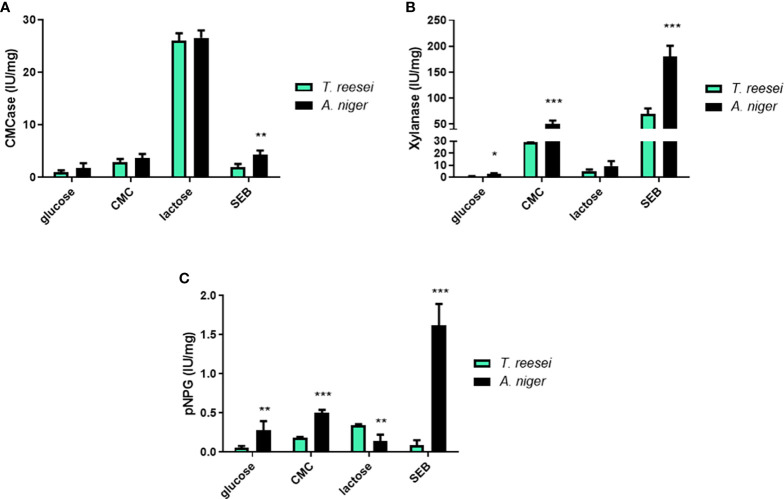
Enzymatic activities for *T. reesei* and *A. niger* grown on glucose, CMC, lactose, and SEB. CMCase **(A)**, xylanase **(B)**, and pNPGase **(C)** activities were normalized by the amount of total secreted protein. The statistical difference between both fungi for the same condition was determined by the two-tailed unpaired Student t-test. *P-value < 0.05; ** <0.01; *** <0.001.

In an attempt to link sugar content and sugar metabolizing enzymes, we next performed enzymatic activity assays for cellulase and xylanase in *T. reesei* and *A. niger* grown on different carbon sources. In glucose, the enzymatic activities for CMCase (**Figure 8A**), xylanase (**Figure 8B**), and pNPGase (**Figure 8C**) were significantly low in both fungi. This result was expected as glucose is considered the repressor sugar of the transcription and formation of hydrolytic enzymes, activating CRE1-mediated catabolic repression (Antoniêto et al., 2016).

In CMC, *A. niger* showed significantly higher xylanase and pNPGase activities compared to *T. reesei* (**Figures 8B, C**). In both fungi, CMCase activities were higher compared to glucose (Student t-test, p-value < 0.05) because cellulose induces the formation of (hemi)cellulases (**Figure 8A**). In *A. niger* grown on SEB, all enzymatic activities were statistically higher than *T. reesei*. CMCase (**Figure 8A**), xylanase (**Figure 8B**) and pNPGase (**Figure 8C**) activities were 2, 2.5, and 18-fold higher in *A. niger*, respectively, confirming the relevant role of bagasse as an inducer for the secretion of industrial enzymes (Yadav et al., 2022). The remarkable difference in pNPG activity is due to the fact that this substrate is specific for β-glucosidases, which are more produced and secreted by this species than *T. reesei* (Srivastava et al., 2019). Commercial *T. reesei*-based cocktails are even supplemented with this class of enzymes, often from *A. niger*, such as the β-glucosidase Novozyme 188 (Novozymes, Denmark) (Champreda et al., 2019). In addition, higher concentrations of glucose and xylose were observed in the supernatant of *A. niger* grown on SEB compared to *T. reesei*, albeit no significant difference was observed between both fungi (**Figure S1**).

Lactose is considered one of the major cellulase inducers on a commercial scale for *T. reesei* (Li et al., 2017), and it has also a positive effect on the production of cellulases and hemicellulases in *A. niger* (Mrudula and Murugammal, 2011; Amore et al., 2013). Thus, as expected, higher CMCase activity was observed for both fungi grown on lactose compared to the other three carbon sources (**Figure 8A**). pNPG activity was statistically higher in *T. reesei* than *A. niger*, corroborating the booster effect of lactose on cellulase formation. Conversely, lactose did not induce a strong xylanase activity for *T. reesei* and *A. niger* as verified for CMC or SEB (**Figure 8B**), but the activity was still much higher than in glucose carbon source (6.8 and 3.6-fold higher in *T. reesei* and *A. niger*, respectively). This phenomenon of greater cellulase activation over hemicellulases by lactose has also been previously observed in the *T. reesei* RUT-C30 strain (Li et al., 2017; Yan et al., 2021).

Finally, the enzymatic activities confirmed the potential of (hemi)cellulase inducer of CMC, lactose, and SEB in both fungi, and also showed that in general *A. niger* had higher (hemi)cellulolytic activities than *T. reesei* in the conditions evaluated in this work (**Figure 8**). Intriguingly, although *A. niger* had higher CMCase and pNPGase activities in CMC, for example, there was no significant difference of growth compared to *T. reesei* (**Figure 1A**). Instead, *T. reesei* accumulated more cellobiose and glucose in this carbon source (**Figure 4**), what could indicate higher formation of cellulases. The relation between metabolites, growth, and enzymatic activity is not a simple equation and several factors might be responsible for the differences observed here, including signal transduction triggered by sugars, transport of molecule inducers, and transcriptional regulation of the enzymes.

### Cross-talk between the metabolomics and transcriptomics

The metabolome profiling of *T. reesei* and *A. niger* revealed alterations in diverse pathways of the primary metabolism, and shed new light on the most abundant metabolites produced in carbon sources of industrial relevance. We next used the transcriptomics data (RNA-Seq or microarray) available in the literature of *T. reesei* and *A. niger* grown on equal or very similar carbon sources (**Tables S4**, **S7**) to explore the regulatory and metabolic mechanisms leading to the observed phenotypes. Although the fungal strains, growth time, and library construction differ among the studies, they provided valuable information which supported the metabolic alterations observed in our study.

Only two compounds from glycolysis lower part, 2-phosphoglycerate and pyruvate, were identified in our experiments (**Table S1**). Accordingly, genes encoding enzymes of the lower glycolytic reactions were among the most expressed in all four carbon sources for both *T. reesei* and *A. niger* (**Figure S3** and **Table S7**). Enolase (jgi|123337, An18g06250) and glyceraldehyde-3-phosphate dehydrogenase (jgi|121593, An16g01830) were found in the top three (except in lactose for jgi|123337) of the most expressed genes of the glycolytic pathway. The latter gene jgi|121593, in particular, was the most expressed in glucose, avicel, lactose, SEB, and even in *T. reesei* grown on wheat straw after 50 h (**Figure S3** and **Table S7**). Glyceraldehyde-3-phosphate dehydrogenase catalyses the reversible formation of 1,3-bisphosphoglycerate and reduces the cofactor NAD^+^ to NADH. Therefore, the substantial expression of this gene in several conditions might be associated with the generation of cofactors that feed the electron transport chain and/or are used as building blocks of lipids, amino acids, and nucleotides biosynthesis (Li et al., 2022). Interestingly, an increase in the metabolite levels and distribution flux of the glycolysis lower part were also observed by Lu et al. (2018), when *A. niger* was grown on glucose in fed-batch cultivations and under oxygen limitation.

Following the carbon flux towards the TCA cycle, five metabolites were identified in the profiles of *T. reesei* and *A. niger*: malate, succinate, aconitate, oxoglutarate, and fumarate, being the first two the most abundant in glucose, CMC, and lactose (in SEB, succinate and fumarate were detected but with low abundance) (**Tables S1**, **S2**). Examining the expression of those 24 and 27 genes belonging to this pathway in *T. reesei* and *A. niger*, respectively, it was possible to observe the transcriptional activation of several genes from both fungi in all substrates (**Figure S3** and **Table S7**). Among them, malate dehydrogenase coding genes (jgi|39524, An15g00070) stood out as the highest expressed genes (**Figure S3** and **Table S7**).

In *T. reesei*, the gene jgi|39524 was the most expressed in lactose and SEB, and had relatively high expression levels in glucose and cellulose. The corresponding gene in *A. niger* (An15g00070) ranked as first in glucose and cellulose, and as second in lactose and SEB (**Figure S3** and **Table S7**). This enzyme is responsible for reversibly converting malate into oxaloacetate and NADH within the TCA cycle, and it is also considered one of the main enzymes of reductive TCA cycle (Gu et al., 2018). Malate dehydrogenase enzymes might be present in the cytosol and mitochondria, and this versatile localization favors the reactions of malate-aspartate shuttle needed for the NAD/NADH cofactor balance between these two cellular compartments (Wang et al., 2011). Although we have not detected oxaloacetate in the metabolomics data, one could speculate that the accumulation of malate (and to a lesser extent of aspartate) (**Table S1**) and the high expression of malate dehydrogenase (**Table S7**) might be linked to the malate-aspartate shuttle and/or oxidative phosphorylation through the balance of NADH.

The glyoxylate shunt pathway could also have contributed to the accumulation of malate and succinate in the samples. This alternative pathway is unleashed under glucose deprivation allowing the cell to use acetate, lactate, fatty acids, and glycerol as carbon sources to feed the gluconeogenesis pathway and produce glucose (Ahn et al., 2016; Chew et al., 2019). It is interesting to notice that the genes encoding isocitrate lyase (jgi|25052, An01g09270) were ranked in the top 10 of the most expressed genes in virtually all carbon sources (**Table S7**). Isocitrate lyase from *T. reesei* (jgi|25052) was the highest expressed gene in lactose, whereas the corresponding gene for *A. niger* (An01g09270) occupied a lower position (**Table S7**). This enzyme converts isocitrate into glyoxylate and succinate (**Figure S3**), and could partially explain the significantly higher accumulation of succinate in the metabolome of *T. reesei* grown on lactose (**Figure 5**, **Table S6**).

The metabolites lactose, galactose, UDP-galactose, galactitol, galactonate, glucitol (sorbitol), and UDP-glucose were identified in the metabolome of *T. reesei* and *A. niger* (**Figure 6** and **Table S1**). In particular, the first six metabolites were found almost exclusively in the lactose samples. Genes encoding aldose-1-epimerase (jgi|24672, An02g09090), UDP glucose-4-epimerase (*gal10*, jgi|137982), galactose-1-phosphate uridylyltransferase (*gal7*, jgi|114842) and phosphoglucomutase (*pgm1*, jgi|107609, An07g06780) were found up-regulated in this carbon source (**Figure S3** and **Table S7**). Their products convert galactose into α-galactose, UDP-galactose into UDP-glucose, galactose 1-phosphate into glucose 1-phosphate, and glucose 1-phosphate into glucose 6-phosphate (G6P), respectively, and these intermediates have the glycolysis as a final destination through the Leloir pathway (Seiboth et al., 2007). In addition, the trehalose-6-phosphate synthase coding genes (jgi|72420, jgi|67350, jgi|24685, An08g10510, An11g10990, An07g08720) were highly expressed in lactose and also in glucose, cellulose, and SEB of both fungi (**Table S7**). This enzyme catalyzes the conversion of UDP-glucose and G6P into trehalose-6-phosphate, the precursor of the metabolite trehalose that was significantly present in *T. reesei* and *A. niger* grown on all four carbon sources (**Tables S1**, **S2**).

GABA was also identified in the metabolome of *T. reesei* and *A. niger* grown on glucose, CMC, and lactose (**Table S1**). GABA might be produced from glutamate by glutamate decarboxylase (**Figure S3**). Its coding gene in *A. niger* (An08g08840) was the most expressed in glucose, ranked as third in lactose and SEB, and as fifth in cellulose (**Figure S3** and **Table S7**). This high expression could partially explain the observed difference in GABA levels in CMC and lactose compared to *T. reesei*, as its corresponding genes (jgi|99110, 109920) were ranked in lower positions. Conversely, the gene encoding glutamine synthetase in *T. reesei* (jgi|101940) was the top expressed gene in glucose and SEB and the top two in lactose and cellulose, while the corresponding genes in *A. niger* (An14g01460, An15g01850) had a low expression in glucose, cellulose, lactose or SEB (**Table S7**). Glutamine is the metabolite formed by glutamine synthetase and the high expression of the gene jgi|101940 corroborates the significant difference of glutamine in *T. reesei* grown on glucose and lactose compared to *A. niger* (**Figure 6** and **Table S7**).

It is worth mentioning that although it has been reported that the transcript levels do not necessarily reflect the metabolic flux in eukaryotic cells (Schwender et al., 2014), it is still possible to retrieve some valuable information from the combination of different ‘omics’ approaches. The transcriptomics datasets of *T. reesei* and *A. niger* available in the literature supported the metabolomics investigated in this study and endorsed some of the metabolic responses employed by both fungi. Nevertheless, flux analysis and metabolic modeling, for example, could be beneficial to fulfill metabolic gaps and give a more accurate picture of their metabolism in the carbon sources used here.

## Conclusions

The industrial fungi *T. reesei* and *A. niger* showed interesting similarities concerning their metabolic composition in all growth conditions analysed. The osmolytes mannitol and trehalose and the amino acids glutamate, glutamine, and alanine were the most abundant metabolites shared by both fungi and seem to be extremely important for the growth and adaptation of *T. reesei* and *A. niger* in simple and complex carbon sources.

Specific differences were observed between both species, which indicate the particular mechanisms adopted by each fungus to survive and cope with stresses under the analysed conditions. *Trichoderma reesei*, for example, accumulated more intermediates from lactose catabolism when grown on lactose, and it might have adopted glyoxylate shunt as an alternative pathway to the TCA cycle for generating energy and glucose through gluconeogenesis. In contrast, *A. niger* concentrated more glycerol and GABA in its cells to be supposedly used as reserve and stress metabolites, respectively. In SEB, *A. niger* showed higher activities for cellulase and hemicellulase, which may have contributed to the activation of the glycolytic pathway as observed at the metabolite level. The transcriptomics data available in the literature corroborated various metabolites identified in this study, and the integration of both approaches has proven to be promising for further studies. However, due to the myriad of routes that a metabolite can follow, it is important to mention that a flux distribution, for example, could help to confirm the assumptions of this work. Furthermore, our findings reaffirm the importance of metabolites of known function in the fungal metabolism and open news perspectives for the elucidation of the role of other metabolites still unexplored. Future investigations might consider the metabolic responses disclosed by this study aiming to develop hyperproducer and/or more robust microbial cell factories.

## Data availability statement

The original contributions presented in the study are included in the article/**Supplementary Material**. Further inquiries can be directed to the corresponding author.

## Author contributions

GB contributed to conception, design of study, investigation, funding acquisition, and wrote the first draft of the manuscript. JO contributed to conception, design of study, supervision and funding acquisition. All authors contributed to the article and approved the submitted version.

## Acknowledgments

We would like to thank Dr. Maurício Luís Sforça and Dr. Silvana Aparecida Rocco from the NMR facility (LNBio – CNPEM, Campinas-SP, Brazil) for all support with the NMR sample preparation, spectrum data processing, and analyses. We are grateful to technician Msc. Lúcia Daniela Wolf and the analytical facility of LNBR-CNPEM, Campinas-SP, Brazil, for the HPLC analyses, and to the technician Bsc. Aline Tieppo de Souza and Rebeca Mariano for support during the experiments. This research has received funding from the Brazilian agencies FAPESP (Grant number 2015/08222-8, 2017/18987-7, and 2022/00474-1), CNPq (Grant number 141574/2015-1), and CAPES.

## Conflict of interest

The authors declare that the research was conducted in the absence of any commercial or financial relationships that could be construed as a potential conflict of interest.

## Publisher’s note

All claims expressed in this article are solely those of the authors and do not necessarily represent those of their affiliated organizations, or those of the publisher, the editors and the reviewers. Any product that may be evaluated in this article, or claim that may be made by its manufacturer, is not guaranteed or endorsed by the publisher.
