# Supplementary material for: Assessing the intracellular primary metabolic profile of Trichoderma reesei and Aspergillus niger grown on different carbon sources
Source: Front Fungal Biol. 2022 Sep 27;3:998361. doi: 10.3389/ffunb.2022.998361 (PMC10512294; doi:10.3389/ffunb.2022.998361)
Supplement: Supplementary file 8 [file Image_1.pdf]

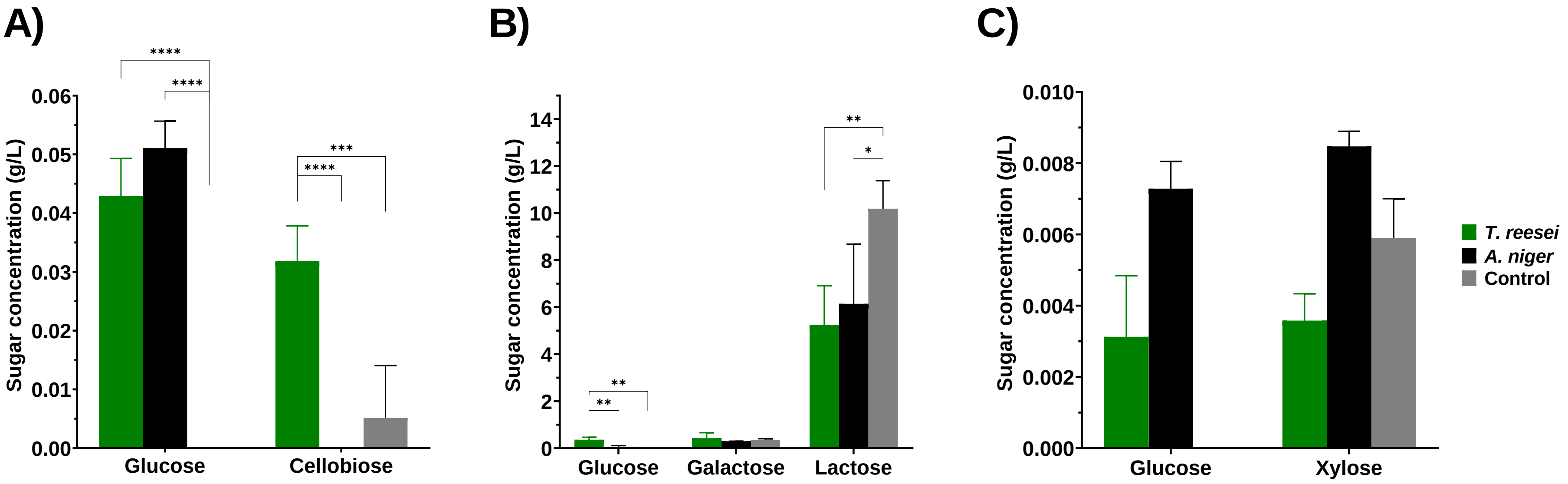

Figure S1. Quantification of sugars in the supernatant of *T. reesei* and *A. niger* grown on CMC (A), lactose (B) and SEB (C), and in the culture media (negative control) after 48 h. Glucose, cellobiose, galactose, lactose and xylose concentrations were measured across the supernatant samples. Glucose was not detected in the CMC control, lactose control and SEB control samples, while cellobiose was not found in the supernatant of *A. niger* grown on CMC and in SEB samples. Concentration of glucose sugar was also measured on glucose carbon source but it was depleted after 48 h for both fungi. Significant difference was verified with one-way ANOVA and Tukey test (****, $p < 0.0001$; ***, < 0.001; **, < 0.01; * < 0.1). No statistical test was applied for SEB samples as the direct comparison between both fungi is not possible due to growth differences in bagasse (please see methods).
