# Supplementary material for: Assessing the intracellular primary metabolic profile of Trichoderma reesei and Aspergillus niger grown on different carbon sources
Source: Front Fungal Biol. 2022 Sep 27;3:998361. doi: 10.3389/ffunb.2022.998361 (PMC10512294; doi:10.3389/ffunb.2022.998361)
Supplement: Supplementary file 9 [file Image_2.pdf]

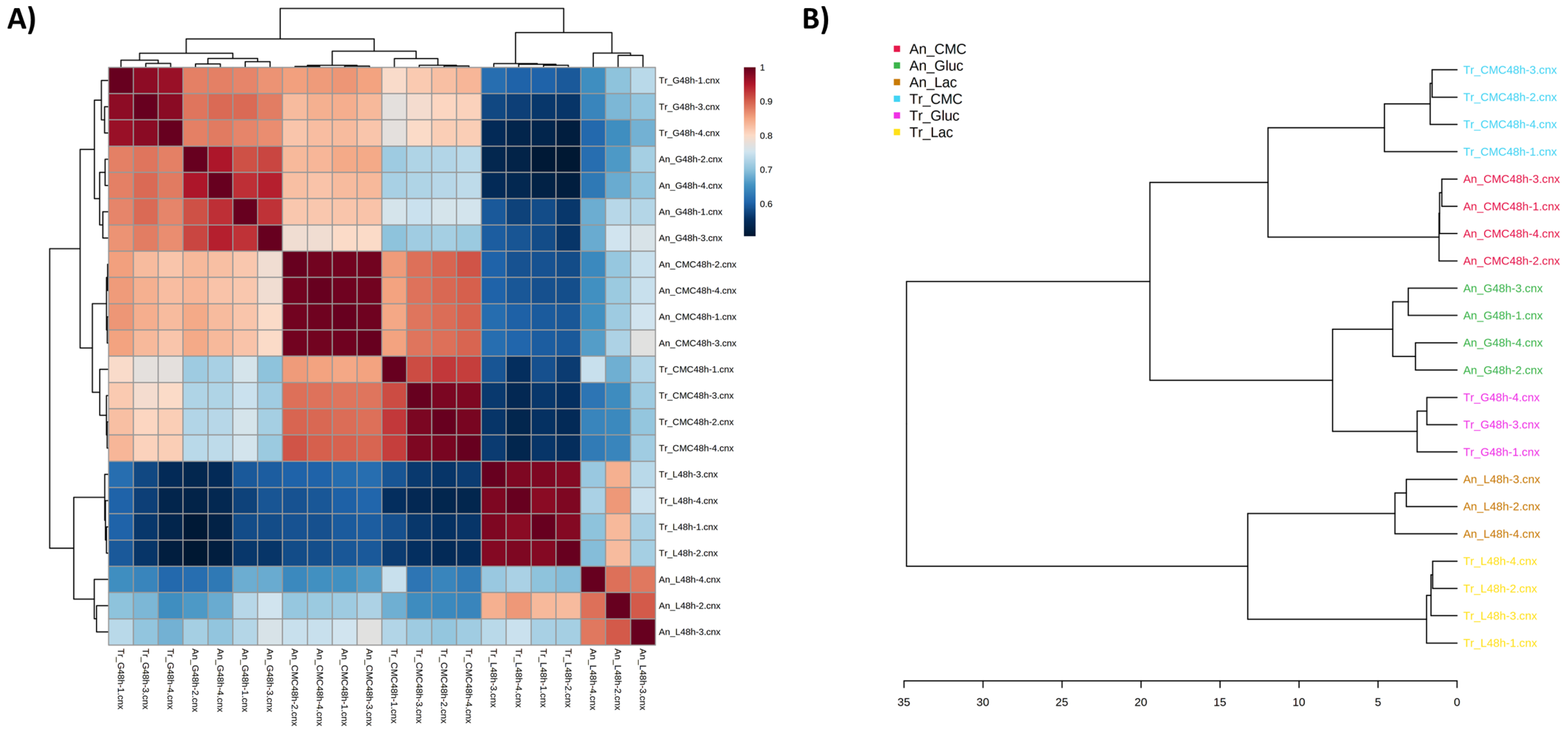

Figure S2. Comparison between the metabolomes of *T. reesei* (Tr) and *A. niger* (An) grown on glucose (G), CMC and lactose (L) after 48 h. A) Heatmap of the Pearson correlation and B) Hierarchical clustering dendrogram of the biological replicates without the two outliers identified (please see methods).
