# Supplementary material for: Assessing the intracellular primary metabolic profile of Trichoderma reesei and Aspergillus niger grown on different carbon sources
Source: Front Fungal Biol. 2022 Sep 27;3:998361. doi: 10.3389/ffunb.2022.998361 (PMC10512294; doi:10.3389/ffunb.2022.998361)
Supplement: Supplementary file 10 [file Image_3.pdf]

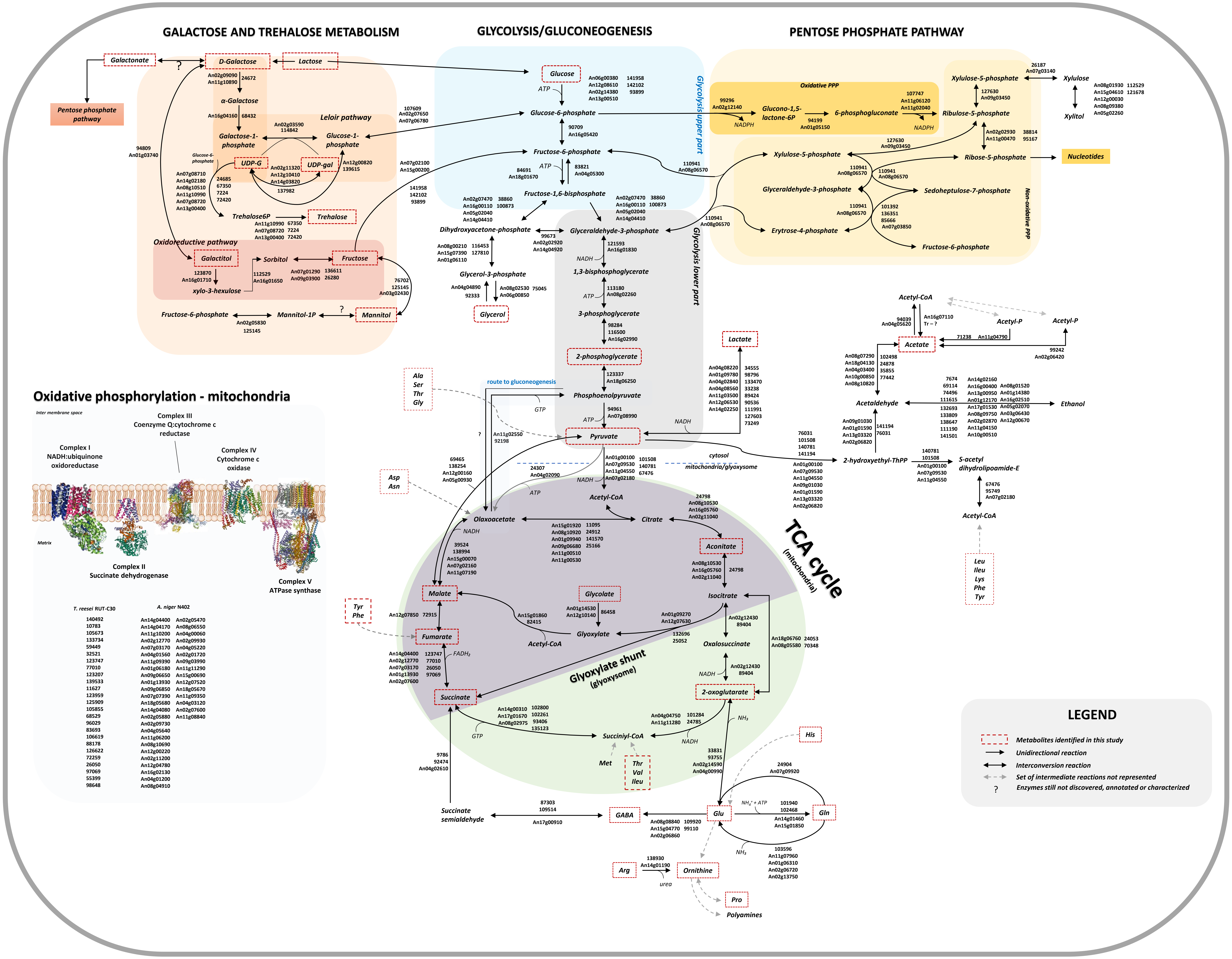

Figure S3. Main metabolic pathways of *T. reesei* RUT-C30 and *A. niger* N402 strains and their corresponding metabolic enzymes. For simplification purpose, some reactions with their substrates/products and metabolic enzymes are not shown. Consequently, the entire metabolic pathways are not completely depicted. Metabolites and enzymes annotation were retrieved from KEGG and JGI databases.
